# Lysosomal cholesterol accumulation in macrophages leading to coronary atherosclerosis in CD38^−/−^ mice

**DOI:** 10.1111/jcmm.12788

**Published:** 2016-01-28

**Authors:** Xiaoyang Xu, Xinxu Yuan, Ningjun Li, William L. Dewey, Pin‐Lan Li, Fan Zhang

**Affiliations:** ^1^ Department of Pharmacology & ToxicologyMedical College of VirginiaVirginia Commonwealth UniversityRichmondVAUSA

**Keywords:** CD38, lysosome, second messenger, cholesterol, coronary atherosclerosis

## Abstract

The disruption in transportation of oxLDL‐derived cholesterol and the subsequent lipid accumulation in macrophages are the hallmark events in atherogenesis. Our recent studies demonstrated that lysosomal Ca^2+^ messenger of nicotinic acid adenine dinucleotide phosphate (NAADP), an enzymatic product of CD38 ADP‐ribosylcyclase (CD38), promoted lipid endocytic trafficking in human fibroblast cells. The current studies are designed to examine the functional role of CD38/NAADP pathway in the regulation of lysosomal cholesterol efflux in atherosclerosis. Oil red O staining showed that oxLDL concentration‐dependently increased lipid buildup in bone marrow‐derived macrophages from both wild type and CD38^−/−^, but to a significant higher extent with CD38 gene deletion. Bodipy 493/503 fluorescence staining found that the deposited lipid in macrophages was mainly enclosed in lysosomal organelles and largely enhanced with the blockade of CD38/NAADP pathway. Filipin staining and direct measurement of lysosome fraction further revealed that the free cholesterol constituted a major portion of the total cholesterol segregated in lysosomes. Moreover, *in situ* assay disclosed that both lysosomal lumen acidity and the acid lipase activity were reduced upon cholesterol buildup in lysosomes. In CD38^−/−^ mice, treatment with Western diet (12 weeks) produced atherosclerotic damage in coronary artery with striking lysosomal cholesterol sequestration in macrophages. These data provide the first experimental evidence that the proper function of CD38/NAADP pathway plays an essential role in promoting free cholesterol efflux from lysosomes and that a defection of this signalling leads to lysosomal cholesterol accumulation in macrophages and results in coronary atherosclerosis in CD38^−/−^ mice.

## Introduction

It is well known that the hallmark event in the pathogenesis of atherosclerosis is lipid accumulation in macrophages. In atherosclerotic lesions, oxLDL is endocytotically taken into macrophages and trafficked to lysosomal organelles, where the endocytosed oxLDL is hydrolysed to free cholesterol by lysosomal acid lipase. Under normal lysosomal function, the generated free cholesterol could be actively transported out of lysosomes to the cytosol and then follows three distinct paths to their destinations: incorporation into cell membrane, re‐esterification with fatty acids by acyl‐CoA cholesterol acyltransferase‐1 and stored as cytoplasmic extra endo/lysosomal inclusions 1, 2, 3, or removal from macrophages with the facilitation of high‐density lipoprotein 4. In atherosclerotic lesions, lipid‐laden lysosomes and re‐esterified cholesterol‐contained lipid droplets could be differentiated under electron microscopy as single membrane–bounded electron‐dense structures and hollowed vacuoles, respectively 5, 6. Extensive studies have been conducted on the mechanisms mediating the aberrant cholesterol intracellular trafficking with the aim to elucidate the lipid deposition in macrophages during atherosclerosis. However, most studies were largely focused on the post‐lysosomal storage of cholesterol and its after‐lysosome transportation.

Since lysosomes serve as the determinant metabolic organelles in hydrolysing oxLDL and locate in the very upstream of free cholesterol intracellular trafficking, it is obligatory to examine the effects of lysosomal cholesterol buildup on macrophage lipid homeostasis. In this regard, there were studies suggesting that the accumulated lipid coexisted in the endo/lysosomes as free cholesterol and cholesteryl ester 5, 7, 8, 9. Consistently, the development of macrophage lysosomal lipid segregation had been shown comprising two distinct consecutive phases, namely a primary accumulation of free cholesterol in the initial phase followed by a late phase of cholesteryl ester deposition 10, 11. It is obvious that further elucidating the regulation of lysosomal cholesterol accumulation will instill a novel insight into the understanding of the macrophage lipid accumulation in the pathogenesis of atherosclerosis during hypercholesterolaemia.

There was evidence that macrophage lipid buildup during atherosclerosis had the features of acquired lysosomal storage disorders 12 such as mucolipidosis type IV, a disease characterized by insufficient lysosomal Ca^2+^ release *via* transient receptor potential mucolipin‐1 channel (TRP‐ML1) and accumulation of phospholipids, sphingolipids and acid mucopolysaccharides in lysosomes 13, 14, 15. Our recent study demonstrated that lysosomal TRP‐ML1‐released Ca^2+^ played a critical role in facilitation of lipids endocytic trafficking and that the Ca^2+^ messenger of nicotinic acid adenine dinucleotide phosphate (NAADP) could profoundly promote this process in prevention of lipid accumulation in lysosomes 16. Nicotinic acid adenine dinucleotide phosphate is a potent intracellular Ca^2+^ second messenger that participates in a variety of pathophysiological processes by releasing Ca^2+^ from lysosomes 17, 18, 19, 20. This nucleotide signalling molecule is mainly produced through an enzyme, CD38 ADP‐ribosylcyclase (CD38), by catalysing the exchange of nicotinamide group from nicotinamide adenine dinucleotide phosphate with nicotinic acid 19, 21, 22, 23, 24. Given the similar features of lysosomal lipid accumulation between atherosclerosis and inherited lysosomal storage disorders as well as the critical role of lysosomal Ca^2+^ release in trafficking lysosomal lipids, it is plausible to speculate that the deficiency of lysosomal Ca^2+^ release by NAADP may lead to insufficient free cholesterol efflux from lysosomes and result in macrophage lipid segregation and atherogenesis.

This study is designed to test the hypothesis that the CD38‐NAADP signalling pathway plays a critical role in removal of free cholesterol from lysosomes in macrophages and that the abnormalities in such CD38‐associated lysosome regulation may contribute to the lysosomal cholesterol accumulation and the pathogenesis of atherosclerosis. Our results demonstrated that the free cholesterol egression from lysosomes was profoundly attenuated in the macrophages with deletion of CD38 gene, which resulted in the lysosomal cholesterol accumulation and atherosclerosis.

## Materials and methods

CD38‐knockout mice (CD38^−/−^, with C57BL/6J background) and C57BL/6J control mice (wild type) were obtained from Jackson laboratory; Western diet (gm%: protein 20, carbohydrate 50 and fat 21) was from Research Dyets, Inc, and all animal experimental protocols were reviewed and approved by the Institutional Animal Care and Use Committee of Virginia Commonwealth University. The mice were housed at 22°C on a 12 hrs light/dark cycle, *ad libitum* to food and water. The reagents and analysis kits are commercial products as following: lysosome enrichment kit, cholesterol quantitation kit, nicotinamide, PPADS and BAPTA‐AM (Sigma‐Aldrich; St. Louis, MO, USA); Bodipy 493/503, Alexa Fluor‐594 chicken anti‐rat IgG (Life Technologies; Grand Island, NY, USA); 4‐methylumbelliferyl palmitate, NED‐19, CD38 goat polyclonal antibody and lysosome‐associated membrane protein 1 (LAMP‐1) rat monoclonal antibody (Santa Cruz Biotechnology, Inc. Dallas, TX, USA); mouse full‐length CD38 constructs (accession number: NM_007646.2), CD38 siRNA (OriGene Technologies, Inc.; Rockville, MD, USA); GenMute and GenJet (SignaGen Laboratories; Rockville, MD, USA), oxLDL (oxidized low‐density lipoprotein) and Dil‐oxLDL [1,1′‐dioctadecyl‐3,3,3′,3′‐tetramethylin dicarbocyanine (Dil)‐labelled oxLDL] (Alfa Aesar; Ward Hill, MA, USA); rabbit antimouse CD68 antibody (Bioss Inc.; Woburn, MA, USA); and oil red O staining kit (American Mastertech Scientific; Lodi, CA, USA).

### Primary culture of bone marrow‐derived macrophages and cell treatments

Mouse bone morrow–derived macrophages were cultured according to the published methods 25, 26. The identity of differentia‐ted macrophages was confirmed by CD68 positive immunostaining. The differentiated macrophages were gently scraped to make a subculture and 12 hrs later used for different experiments as described below.

### Transfection or silencing of CD38 gene in macrophages

CD38 siRNA and the full length CD38 cDNA plasmid were transfected into macrophages with GenMute and GenJet, respectively. The changes of CD38 protein levels were confirmed by Western blot analysis 24 hrs after gene intervention. Different inhibitors of CD38/NAADP signalling pathway including nicotinamide (6 mM), PPADS (50 μM) and NED‐19 27 (10 μM) were applied to wild‐type macrophages 1 hr prior to the addition of oxLDL at a final concentration of 30 μg/ml or Dil‐oxLDL of 5 μg/ml. The analysis of lipid accumulation in macrophages was conducted in oxLDL‐treated groups 24 hrs later after incubation. For Dil‐oxLDL groups, Dil‐oxLDL red fluorescence was examined with confocal microscopy after 2 hrs, 37°C incubation 28. In CD38 gene‐silenced wild‐type macrophages or CD38 rescued CD38^−/−^ cells, the oxLDL treatment was followed 48 hrs later after these gene manipulations. The delivery of NAADP (100 nM) to the CD38^−/−^ macrophages was fulfilled using an ultrasound microbubble method as described previously 16, 22.

### CD38^−/−^ mouse atherosclerosis model and heart sections

CD38^−/−^ and wild‐type male mice at 8‐week age were randomly assigned to Western diet and normal diet groups and maintained on these diets for 12 weeks to establish atherosclerosis model. Before being sacrificed for tissue collections, the mice were fasting overnight. While deeply anaesthetized with pentobarbital at 50 mg/kg i.p., the blood was taken for cholesterol biochemical assay followed by *in situ* heart PBS perfusion, heart collection and aorta dissection. The isolated hearts were proceeded to cryostat cardiac sections at 8 μm thickness and used for immunostaining and oil red O detection of atherosclerotic lesions or underwent paraffin embedding to cut slides for HE examination of artery morphology.

### HPLC analysis of NAADP conversion rate in macrophages

In determination of CD38‐associated NAADP production in macrophages, a base‐exchange related NAADP conversion rate was analysed by HPLC assay as we described previously 19, 22. Nicotinic acid adenine dinucleotide phosphate production was quantified in macrophages from wild‐type, CD38^−/−^ and CD38^−/−^ with CD38 gene transfection.

### Examinations of lipid deposition in macrophages and coronary artery wall by oil red O staining and transmission electron microscopy

The lipid deposition in macrophages and coronary atherosclerotic lesions was identified using oil red O staining kit by following the manufacturer's manual. The oil red O images were taken with transmitted light microscopy to morphologically examine lipid deposition in macrophages and atherosclerotic lesions in the wall of coronary artery. In macrophages, the stained oil red O was further extracted with isopropanol, and the extractions were subject to spectrometrical measurement to quantify the deposited lipids 29. In coronary artery sections, the size of atherosclerotic lesions was analysed with Image‐Pro premier software (Media Cybernetics; Rockville, MD, USA). Transmission electron microscopy examination of lipid accumulation in macrophages and mouse coronary arteries were performed according to published methods 30, 31.

### Differentiation of lysosome‐compartmentalized cholesterol accumulation in macrophages

Twenty‐four hours after oxLDL incubation, the macrophages were stained with Bodipy 493/503, a fluorescent neutral lipid dye, at a concentration of 2.5 μM to reveal the overall lipid droplets in macrophages as previously described 32. The buildup of free cholesterol in macrophages was disclosed by filipin staining at 50 μg/ml 5, 33. Lysosomal organelles were identified with immunostaining LAMP‐1 and secondary antibody coupled with Alexa fluor 594 by the method detailed in our previous studies 32. The confocal microscopy images were taken using an Olympus Fluoview System (Olympus; Melville, NY, USA), which consists of an Olympus BX61WI inverted microscope with an Olympus Lumplan F1 ×60, 0.9 numerical aperture, and oil‐immersion objective at λEx/λEm (nm) of 350/450, 493/503 and 595/615 for imaging free cholesterol, lipid and LAMP‐1, respectively. The lysosome‐located lipid and free cholesterol as well as the extent to which they were trapped in lysosomes were dissociated by colocalization analysis with lysosomal LAMP‐1 immunostaining using Image‐Pro Premier as we described previously 16.

### Analysis of lysosomal lumen pH and cholesteryl ester hydrolase activity by fluorescence microplate reader

A dual‐emission ratiometric measurement in lysosomal lumen pH was adopted using LysoSensor Yellow/Blue dextran dye as described in publication 34. In brief, a pH calibration standard curve was set up by microplate reader measuring LysoSensor fluorescence emission ratio λEm530/Em450 at λEx360 (nm) from lysosomes with series of manipulated lumen pH at 3.5, 4.5, 5.5, 6.5 to 7.5. The LysoSensor fluorescence ratio readings from oxLDL‐challenged macrophages were converted to pH by relating to the pH standard curve. The lysosomal cholesterol ester hydrolase activity was determined by measuring the conversion rate of 4‐methylumbelliferyl palmitate substrate to the fluorogenic molecule of 4‐methylumbelliferone according to the methods detailed in the publications 35, 36. Briefly, 24 hrs after incubation with oxLDL, the macrophages in 96‐well plate were treated with the substrate of 4‐methylumbellifeyl palmitate at a concentration of 0.1 mM for 1 hr, 37°C, 5% CO_2_. The hydrolysed fluorescence product of 4‐methylumbelliferone was then measured at (nm) λEx/Em: 360/450.

### Macrophage lysosome fraction and biochemical analysis of lysosomal cholesterol

Lysosome organelles from oxLDL‐treated wild and CD38^−/−^ macrophages were isolated by gradient centrifugation using lysosome enrichment kit as we did previously 16, 37, 38. In brief, macrophages were broken with a Dounce homogenizer, and the cell homogenates were overlaid on the top of multilayer OptiPrep gradients (in %) 17, 20, 23, 27, 30. After ultracentrifuge at 4°C, 145,000 × g, 2 hrs, the lysosome‐containing layer was collected and subjected to PBS wash to rid of Optiprep. The purity of lysosome was confirmed by lysosome acid phosphatase activity assay (Acid phosphatase assay kit; Sigma‐Aldrich), as well as NADPH‐cytochrome c reductase and alkaline phosphodiesterase activity measurement to ensure free plasma membrane and endoplasmic reticulum contamination. The lysosomal cholesteryl ester and free cholesterol were measured by cholesterol quantitation kit with the procedures described in user's manual.

### Confocal microscopic detection of lysosomal free cholesterol and macrophages in coronary artery

Filipin staining free cholesterol and CD68 immunostaining were performed according to published methods 5, 33 with minor modifications. In brief, mouse coronary artery frozen sections were fixed with 4% paraformaldehyde (PFA) in PBS for 20 min. at room temperature (RT). The PFA was then quenched with 0.3 M glycine in PBS for 10 min. For free cholesterol staining, the artery frozen sections were incubated with 50 μg/ml filipin for 1 hr at RT. The free cholesterol was detected by confocal microscopy image of filipin at (nm) λEx/Em: 350/450. For immunostaining macrophage marker protein of CD68 39, rabbit antimouse CD68 was incubated with the artery tissue section at 1:100, then conjugated with Alexa Fluor 555‐labelled donkey anti‐rabbit IgG (dilution 1:300). The CD68/Alexa Fluor 555 image was acquired at (nm) λEx/Em: 555/565.

### Statistics

Data are presented as mean ± S.E. Significant differences between and within multiple groups were examined using anova for repeated measures, followed by Duncan's multiple‐range test. Student's *t*‐test was used to evaluate the significance in differences between two groups of observations. *P* < 0.05 was considered statistically significant.

## Results

### NAADP production in macrophages

Using established NAADP HPLC analysis, NAADP conversion rate was found to be lacking in CD38^−/−^ macrophages compared with that in wild‐type cells (0.003 ± 0.001 *versus* 0.133 ± 0.012 nmol/min./mg protein, *n* = 5, *P* < 0.05). However, after CD38 gene was rescued by transfection of its gene construct, the NAADP production in CD38^−/−^ was restored to the level detected in wild‐type cells.

### Disruption of CD38/NAADP signalling leads to lysosomal lipid deposition in macrophages

**Figure 1:**
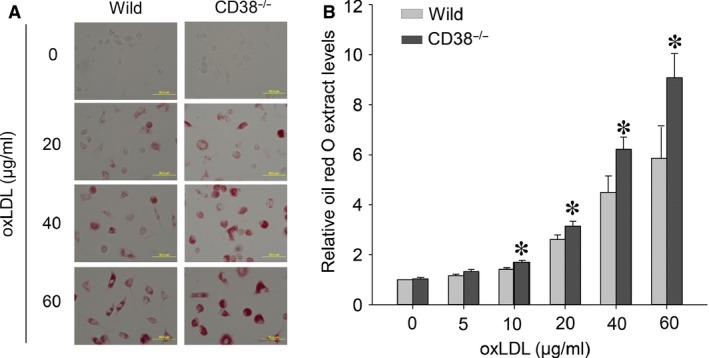
Confirmation of CD38 genotype‐associated lipid deposition in mouse bone marrow–derived macrophages. (**A**) Transmitted light microscopy images showed oil red O–stained wild‐type (wild) and CD38^−/−^ macrophages after exposed to serial concentrations of oxLDL, 24 hrs. (**B**) Normalized spectrometric measurements of isopropanol extractions from oil red O–stained macrophages (*P < 0.05, significant differences from wild‐type cells within the same oxLDL concentrations, *n* = 5 batches of macrophages).

**Figure 2:**
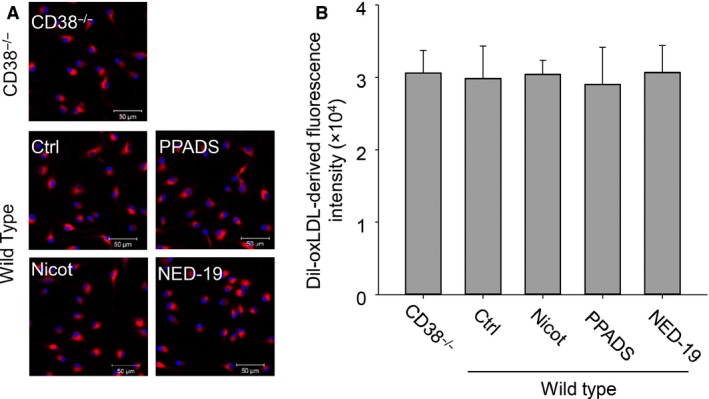
Disruption of CD38/NAADP signalling pathway has no effects on oxLDL uptake rate in CD38^−/−^ and wild‐type macrophages. (**A**) Confocal microscopy images of macrophages treated with Dil‐oxLDL. Red: Dil‐oxLDL derivatives, blue: DAPI‐stained nuclei. (**B**) Quantification of Dil‐oxLDL‐derived red fluorescence intensity from CD38^−/−^ macrophages and wild‐type macrophages with CD38/NAADP pathway inhibitors of Nicot (Nicotinamide), PPADS (Pyridoxal‐phosphate‐6‐azophenyl‐2′,4′‐disulfonic acid) and NED‐19 (*n* = 5).

To investigate the effects of CD38/NAADP signalling on macrophage lipid buildup, we first compared the lipid accumulation profile between wild and CD38^−/−^ macrophages challenged with oxLDL. We found that oxLDL from (in μg/ml) 0 to 60 could concentration‐dependently increase lipid deposition in these two types of cells, but with more lipid accumulation in CD38^−/−^ macrophages as visualized by brighter red images from oil red O staining (Fig. 1A) and significantly higher normalized spectrometric readings from isopropanol extractions of oil red O–stained cells (Fig. 1B). To clarify whether disruption of CD38/NAADP pathway has effects on the rate of oxLDL uptake, we applied Dil labelled‐oxLDL (Dil‐oxLDL), a red fluorescent derivative of oxLDL, to CD38^−/−^ macrophages and wild‐type cells in the presence of different CD38/NAADP signalling inhibitors. No difference in red brightness from confocal microscopy images was observed among different group cells (Fig. 2A). The qualified red fluorescence intensity also showed no disparity among different groups (Fig. 2B).

**Figure 3:**
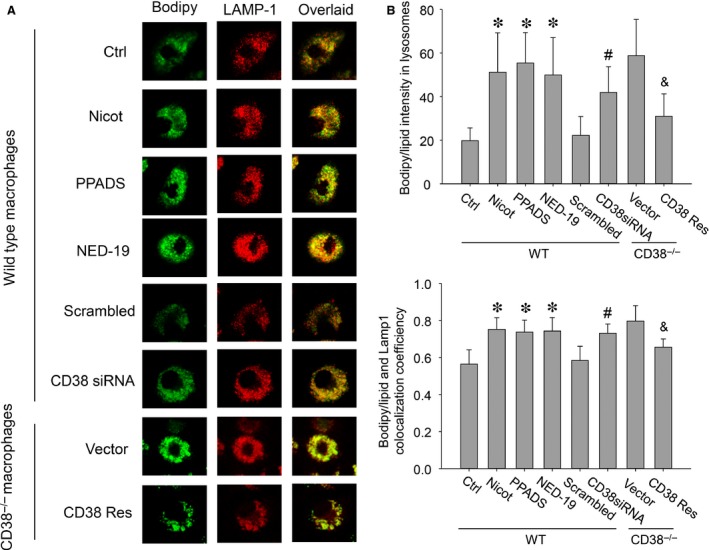
Lysosome‐compartmentalized lipid upon CD38/NAADP signalling disruption constitutes a major portion of the total lipid deposited in macrophages. (**A**) Confocal microscopy images showed Bodipy‐stained lipid (Bodipy, green), immunostaining lysosome marker protein of LAMP‐1 (LAMP‐1, red). Yellow spots in the overlaid images represented the lipid segregation in lysosomes; (**B**) intensity analysis of Bodipy‐stained lipid in lysosomes; (**C**) colocalization efficiency of lysosome organelles and the overall lipid deposited in macrophages (*P < 0.05 *versus* Ctrl, ^#^ *P* < 0.05 *versus* Scrambled, ^&^ *P* < 0.05 *versus* Vector; *n* = 5).

Since that deficiency of lysosomal Ca^2+^ release pathologically attributes to the lysosome lipid deposition in mucolipidosis type IV disease and that intracellular lipid buildup during atherosclerosis has the characteristics of acquired lysosomal storage disorders, we thereby proceeded to inspect lysosomal lipid accumulation in CD38/NAADP Ca^2+^ signalling disrupted macrophages. Western blot confirmation of CD38 siRNA interference efficiency was presented as Figure S1. Bodipy 493/503 staining results showed that in wild‐type macrophages, all CD38/NAADP pathway inhibitors rendered a profound increase of lipid deposition in lysosomes as visualized from brighter green confocal images (Fig. 3A) and measured by the Bodipy fluorescence intensity (Fig. 3B). Consistently, in CD38^−/−^ macrophages, lysosomal lipid deposition was also dramatically enhanced compared with that in wild‐type control cells. The enhanced lysosomal lipid deposition was markedly attenuated after CD38 gene transfection (Fig. 3A and B). Furthermore, the colocalization efficiency between lysosome‐compartmentalized lipid and the overall deposited lipid in either wild‐type or CD38^−/−^ macrophages was correlated with the extent of lipid buildup in lysosomes (Fig. 3C).

### Free cholesterol constitutes a major portion of the total cholesterol segregated in lysosomes

**Figure 4:**
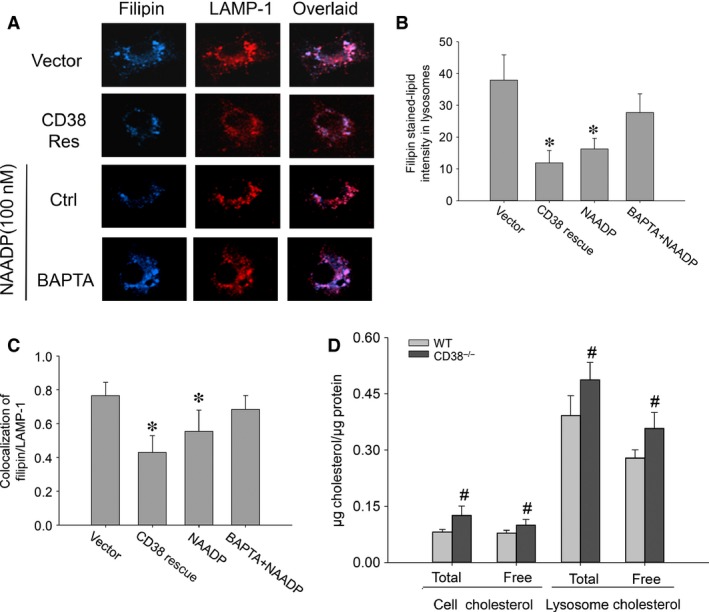
Rescuing CD38/NAADP signalling pathway attenuates free cholesterol accumulation in lysosomes of CD38^−/−^ macrophages on oxLDL. (**A**) Confocal microscopic images showed filipin‐stained‐free cholesterol (Filipin, blue) and immunostaining lysosomal LAMP‐1 (red). Purple spots in the overlaid image represented the free cholesterol sequestered in lysosomes; (**B**) quantification of free cholesterol intensity in lysosomes of CD38^−/−^ macrophages; (**C**) colocalization efficiency of lysosome organelles and the overall deposited free cholesterol; (**D**) cholesterol levels of lysosomal fractions between wild and CD38^−/−^ macrophages (*P < 0.05 *versus* Vector, ^#^ *P* < 0.05 total and free cholesterol in CD38^−/−^ macrophages or lysosomes *versus* their counterparts from wild‐type macrophages, *n* = 5).

Given cholesterol egression from lysosomes is a Ca^2+^‐dependent process and free cholesterol is the only cholesterol form that could be transported out of lysosomes, we further defined the free cholesterol portions in lipid inflicted lysosomes. Filipin staining showed that the deposited free cholesterol in lysosomes of CD38^−/−^ macrophages was significantly diminished with CD38 gene transfection or direct NAADP supplement, while NAADP delivery failed to reduce the lysosomal deposition of free cholesterol in the cells pretreated with Ca^2+^ chelator of BAPTA. In the presence of BAPTA, NAADP‐mediated Ca^2+^ effects were deprived (Fig. 4A and B). Colocalization efficiency analysis of filipin/cholesterol with lysosomal LAMP‐1 revealed that the lysosome‐trapped free cholesterol was reduced in CD38^−/−^ cells with the rescue of CD38/NAADP signalling (Fig. 4C).

Direct quantification of cholesterol from purified lysosomes further dissected the free cholesterol from the total cholesterol sequestered in lysosomes, and the results in Figure 4D showed the accumulations of both free and total cholesterol in CD38^−/−^ macrophage lysosomes were significantly enhanced compared with their counterparts in lysosomes from wild‐type macrophages (Fig. 4D, in μg cholesterol/μg protein, 0.39 ± 0.05 and 0.27 ± 0.02 *versus* 0.48 ± 0.05 and 0.36 ± 0.04, respectively). The enzymatic confirmation of the purity of lysosomal fraction was presented as Figure S2.

### Lysosomal lipid accumulation decreases lysosomal lumen acidity and compromises lysosomal cholesteryl ester hydrolase activity

**Figure 5:**
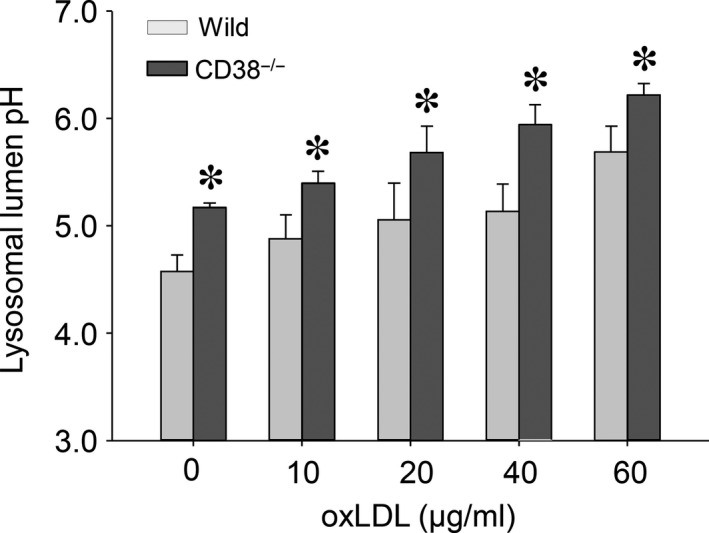
Lysosomal lipid accumulation attenuates lysosomal lumen acidity. *In situ* ratiometric results of lysosomal lumen pH from both wild type and CD38^−/−^ macrophages (*P < 0.05 CD38^−/−^ *versus* wild type within the same oxLDL concentrations, *n* = 5).

The maintenance of an optimal acidic milieu is the precondition to sustain a normal lysosome function. The ratiometric measurement results demonstrated that lysosomal lumen acidity was oxLDL concentration‐dependently decreased in both of wild and CD38^−/−^ macrophages but with more decrements in lysosomes from CD38^−/−^ cells (Fig. 5: in pH, 4.57 ± 0.54, 4.88 ± 0.78, 5.05 ± 1.20, 5.13 ± 0.90 and 5.68 ± 0.84 in lysosomes from wild‐type macrophages *versus* 5.17 ± 0.08, 5.40 ± 0.22, 5.68 ± 0.49, 5.94 ± 0.37 and 6.21 ± 0.22 in lysosomes from CD38^−/−^ macrophages, corresponding to oxLDL concentrations in μg/ml from 0, 10, 20, and 40 to 60).

**Figure 6:**
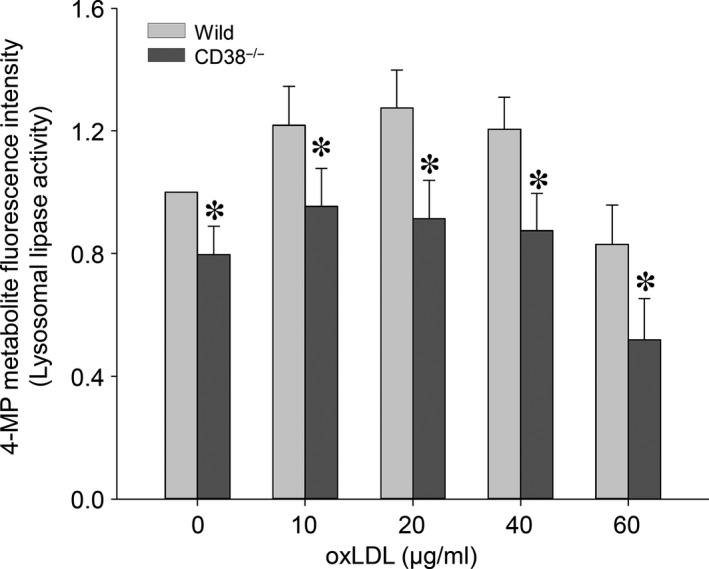
*In situ* measurement of fluorogenic 4‐methylumbelliferone product in lysosomes to show the effects of lysosomal lumen lipid sequestration on lysosomal acid lipase activity in both wild type and CD38^−/−^ macrophages (*P < 0.05 CD38^−/−^ *versus* wild type within the same oxLDL concentrations, *n* = 6).

To elucidate the impacts of lysosome lipid segregation on lysosomal cholesteryl ester hydrolase activity, we measured the production of fluorogenic metabolite of 4‐methylumbelliferone from the hydrolysis of methylumbelliferyl palmitate substrate in lysosomes. The normalized lysosomal 4‐methylumbelliferone fluorescence intensity readings resembled a saddle‐shaped change over the tested different oxLDL concentrations in both wild and CD38^−/−^ macrophages. The lipase activity was increased first and then gradually decreased. This increased activity of lysosomal acid lipase reflects the metabolism reservation of this enzyme. The significant higher measurements were found in wild‐type group across different oxLDL concentrations (Fig. 6).

### Deletion of CD38 gene promotes coronary atherosclerosis in CD38^−/−^ mice

**Figure 7:**
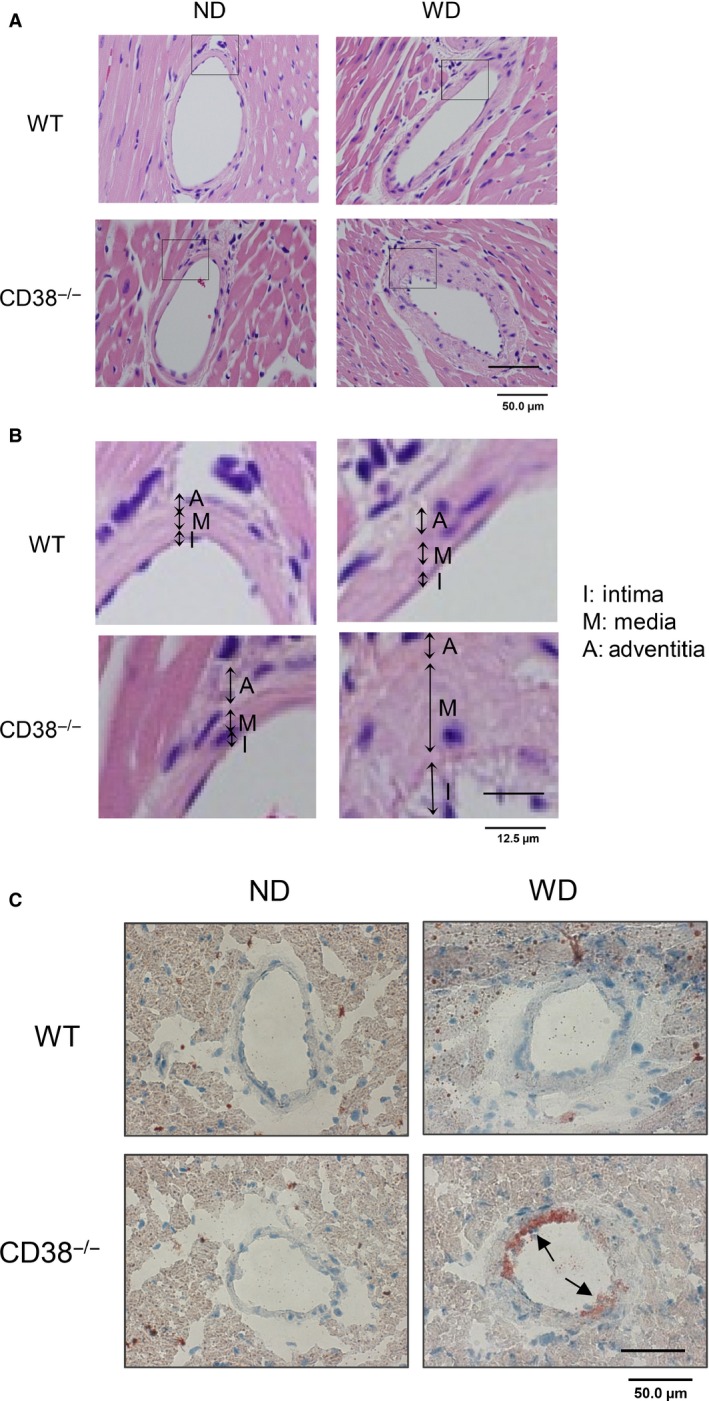
Histological examinations of atherosclerosis in CD38^−/−^ mouse coronary artery wall. (**A**) Light microscopy images of HE staining showed extensive intimal and media layer thickening in the coronary artery wall from CD38^−/−^ mice on Western diet (WD) but not in other groups. (**B**) The squared regions were amplified and the layers of intima, media and adventitia identified (*n* = 5); (**C**) oil red O staining to examine the atherosclerotic lesions in coronary artery. The positive staining was only found from CD38^−/−^+WD mouse group and the area was quantified (in μm^2^) 1298.1 ± 332.4; or the atherosclerotic region represented 21.15 ± 5.12% of whole transverse artery section, *n* = 5. Scale bar: 50.0 μm, applies to all images.

Since the deficiency of CD38/NAADP signalling led to lysosomal lipid accumulation *in vitro*, it is obligated to investigate its proatherogenic effects in CD38 gene abrogated mice. Figure 7A is the transmitted light microscopy images of transverse sections of coronary artery with HE staining. The squared regions were amplified to distinguish the layers of intima, media and adventitia. Obviously, the coronary artery from Western diet‐treated CD38^−/−^ mice had an extensive intimal thickening. The increased thickness could also be seen in the media layer. These morphological features typically resemble an atherosclerosis (Fig. 7B). After oil red O staining, the deposited lipids were found throughout the atherosclerotic lesions in CD38^−/−^ mouse on Western diet but not in other groups of mice (Fig. 7C). It should be noticed that the atherosclerotic lesions in CD38^−/−^ mice on Western diet were only found in the coronary artery but not on the aorta and aorta root as usually observed in the empirical atherosclerotic mouse model of LDLr^−/−^. Also, there were no significant differences in the plasma cholesterol levels between these wild and CD38^−/−^ mice that were fed on either normal or Western diet (Fig. S3).

**Figure 8:**
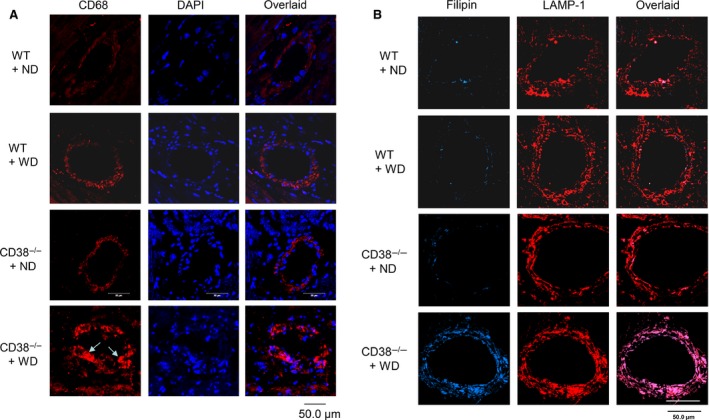
The aggregation of macrophages and deposition of free cholesterol in coronary atherosclerotic lesions from CD38^−/−^ mice on WD. (**A**) Confocal microscopy images of macrophages by immunostaining CD68 (CD68, red) in the coronary artery transverse sections. Much stronger red staining intensity was displayed in the atherosclerotic region from CD38^−/−^ mice on the WD (arrow) compared with others (*n* = 5). (**B**) Confocal microscopy images of free cholesterol deposition in coronary artery wall from CD38^−/−^ mice on the WD (*n* = 5). Scale bar: 50.0 μm, applies to all images.

The macrophage aggregations in atherosclerotic lesions were examined by immuostaining CD68. Consistence with the oil red O staining, the numbers of macrophages were found significantly increased only in the atherosclerotic lesions of coronary artery from CD38^−/−^ mice on the Western diet, but not in other groups (Fig. 8A). The lysosomal accumulation of free cholesterol in the coronary artery wall was also examined by costaining of filipin and anti‐LAMP‐1 antibody. The confocal microscopy images of the fluorescence staining showed that the vessel from CD38^−/−^ mice on the Western diet had the brightest blue spots (filipin‐stained free cholesterol), which were colocalized well with the red stains (immunostaining of lysosomal marker, LAMP‐1) and generated profound purple spots in the overlaid images, suggesting the significant free cholesterol accumulation in lysosomes of coronary arterial cells, but this free cholesterol deposition was not found in the artery wall from wild‐type mice or CD38^−/−^ mice on the normal diet (Fig. 8B).

**Figure 9:**
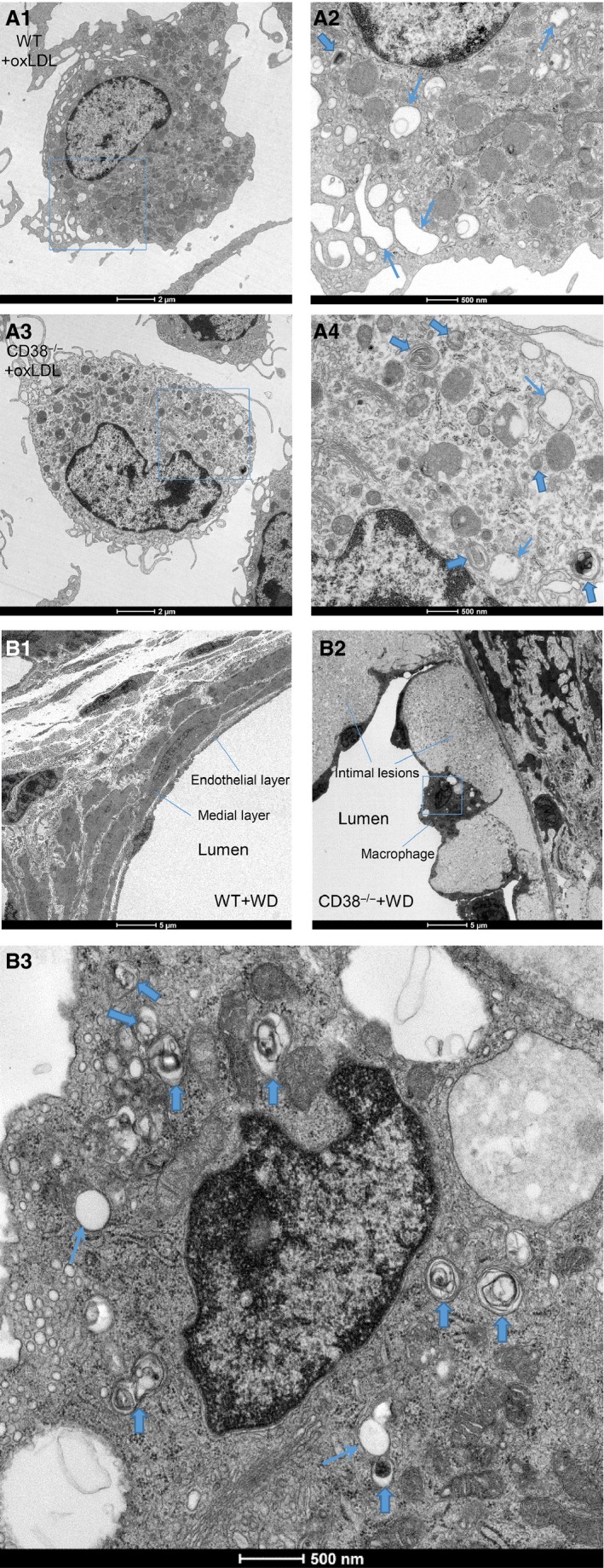
Electron microscopy examination of lipid accumulation in wild type and CD38^−/−^ macrophages on oxLDL in culture and coronary artery from wild and CD38^−/−^ mice fed with Western diet. (**A**) A1, wild‐type macrophage on oxLDL (WT + oxLDL), A2, amplified interesting area from squared portion in A1; A3, CD38^−/−^ macrophage on oxLDL (CD38^−/−^ + oxLDL), A4, amplified interesting area from squared portion in A3. (**B**) B1, normal coronary artery structures from wild‐type mouse fed with Western diet (WT + WD); B2, coronary atherosclerotic lesions form CD38^−/−^ mouse fed with Western diet (CD38^−/−^ + WD), B3, amplified interesting area (squared portion) from lesional macrophage in B2. The accumulation of lipid in cultured CD38^−/−^ macrophage on oxLDL and lesional macrophage from CD38^−/−^ mouse fed with Western diet featured lipid segregation in lysosomes – abundant single membrane–bounded electron‐dense structures and multilamellar inclusions (Bold arrow), but less cytoplasmic lipid droplets (hollowed vacuoles) than in wild‐type macrophage on oxLDL (arrow). Micrograph scales were embedded in the images (*n* = 3).

Using electron microscopy, we examined the profiles of lipid accumulation in both wild‐type and CD38^−/−^ macrophages in culture treated with oxLDL, as well as the coronary artery from wild‐type and CD38^−/−^ mice fed with Western diet. The electron micrographs showed that wild‐type macrophage on oxLDL appeared foamy, a morphology that was mainly derived from cytoplasmic lipid droplets. However, CD38^−/−^ macrophages, from either culture or intimal atherosclerotic lesions, were abundant with multilamellar inclusions and single membrane‐bounded electron‐dense structures, which featured a typical morphology of lipid accumulation in lysosomes. The coronary artery from wild‐type mouse on Western diet showed a normal structure (Fig. 9).

## Discussion

This study has demonstrated that CD38/NAADP Ca^2+^ signalling pathway promotes free cholesterol egression out of lysosomes in macrophages. The deficiency of this CD38‐associated regulation of lysosome function contributes to the lysosomal cholesterol sequestration in macrophages and coronary atherosclerosis in CD38 ^−/−^ mice.

Our HPLC analysis showed that CD38 acted as a predominant enzyme in the production of NAADP in mouse macrophages. This result is consistent with the findings by others that CD38 was responsible for the endogenous NAADP generation in lymphokine‐activated killer cells and pancreatic acinar cells 23, 24, and it also agrees with our previous studies in coronary arteries 19. However, there was a report that no NAADP concentration difference had been found between wild and CD38^−/−^ mice in the examined spleen, heart, uterus and liver tissues 40. It seems that CD38 has tissue specificity in the production of endogenous NAADP.

Our oil red O and Bodipy 493/503 staining results revealed that the segregated lysosomal lipid due to CD38/NAADP deficiency represented a major portion of the totally deposited lipid in macrophages. Moreover, filipin staining and lysosomal fraction analysis unveiled that the free cholesterol constituted a significant fraction of the total cholesterol in lysosomes. It is noteworthy that the feature of lysosome‐dominated lipid accumulation in macrophages is associated with the upstream location of lysosomes in both oxLDL hydrolysis and cholesterol transportation. Our *in situ* pH measurement showed that the compartment acidity in lipid‐filled lysosomes of macrophages was decreased, which is consistent with the reports that accumulated cholesterol in lysosomes has the inhibitory effects on lysosomal V‐H^+^‐ATPase activity 10, 11, a driving force to generate H^+^ gradients across lysosomal membranes and maintain an acidic milieu in lysosomal lumen. In line with the abated lysosomal acidity, we found that lysosomal hydrolysis conversion rate of 4‐Methylumbelliferyl palmitate substrate to the fluorogenic molecule of 4‐methylumbelliferone was reduced. Since the effectiveness of lysosomal acid hydrolase in metabolizing cholesteryl ester depends on an optimal acidity, the decreased lysosomal acidic potency would eventually compromise lysosomal acid lipase efficacy in conversion of esterified cholesterol into free cholesterol 41 and thereby prevent cholesterol egression out of lysosomes, which forms a vicious cycle in cholesterol metabolism and transportation. In addition, the V‐H^+^‐ATPase‐derived H^+^ gradient is also important for coupling Ca^2+^/H^+^ exchange in sequestration of Ca^2+^ into lysosomes 22, 42, and the decreased lysosomal acidity may result in the depletion of Ca^2+^ in lysosomes 43, a critical source of Ca^2+^ for cholesterol transport out of lysosomes. Therefore, the deterrence in free cholesterol transportation out of lysosomes plays a pivotal role in lysosomes by depriving lysosomal normal functions.

The lysosome‐dominated lipid accumulation in CD38^−/−^ was also confirmed by electron microscopy study. Under electron microscope, CD38^−/−^ macrophages from either culture on oxLDL or atherosclerotic lesions displayed multilamellar inclusions and single membrane–bounded electron‐dense structures, which featured lysosomal lipid accumulation. However, lipid segregation in wild‐type macrophages on oxLDL in culture showed a foamy morphology and resembled the well‐studied foam cells from LDLr^−/−^ and ApoE^−/−^ atherosclerotic lesions, which represented the occurrence of cytoplasmic extramural‐lysosome lipid droplets. This electron microscope‐based differentiation of lysosome from lipid droplets in lipid storage has been well documented previously 5, 6, 30. In these wild‐type, LDLr^−/−^ or ApoE^−/−^ macrophages, lysosomal functions in egressing free cholesterol out of lysosomal compartment remained intact. The development of cytoplasmic lipid droplets in these macrophages are largely associated with the impedance of reverse free cholesterol transportation out of cells, a sequential post‐lysosome event that includes neutral lipase hydrolysis of cholesteryl ester, ATP‐binding cassette transporter A1 trafficking cholesterol out of cell to ApoE or high‐density lipoprotein, delivery of these lipoproteins to hepatic SR‐B1 and LDL receptors for finally cleared off in the liver. Therefore, the lack of ApoE, LDLr and HDL rendered a prominent cholesteryl ester deposition in cytoplasm and constituted a significant difference from free cholesterol‐featured lysosomal lipid accumulation.

The free cholesterol–characterized lipid buildup in lysosomes of macrophage in CD38^−/−^ mice may set it apart in atherogenesis. Recent studies have found that deposited free cholesterol and the associated changes in lysosomal functions play a critical role in initiating and sustaining inflammation during atherosclerosis. First, the accumulated free cholesterol is able to form cholesterol crystal 44, and this crystalized cholesterol has been shown to rupture phagolysosomal membrane and cause the activation of inflammasome, which in turn leads to the secretion of inflammatory cytokines including interleukin (IL)‐1β in a cascade reaction 45, 46, 47. Second, the accumulation of cholesterol may cause the cathepsins leakage out of lysosome and release into the cytoplasm. The cytosolic cathepsins can act as cleavage enzymes to initiate apoptosis and contribute to the formation of necrotic core in atherosclerosis, and third, the sequestration of cholesterol in lysosomes may prevent this organelle from receiving *de novo* synthesized lysosomal enzymes and cause the secretions of these enzymes into the interstitial 48. It has been found that lysosomal cathepsins could degrade the extracellular matrix by proteolysis of elastin, collagens and proteoglycans 49, 50. The degradation of extracellular matrix may facilitate the migration and invasion of macrophages into the atherosclerotic lesions. Our recent studies demonstrated that the proinflammatory IL‐1β secretion was significantly increased in oxLDL‐treated CD38^−/−^ macrophages and the plasma IL‐1β markedly elevated in CD38^−/−^ mice on Western diet 51. This proinflammatory propensity upon lysosomal cholesterol accumulation in macrophages may play a synchronic role in the development of atherosclerosis.

Nonetheless, how lysosomal cholesterol accumulation in macrophages renders CD38^−/−^ mouse an atherosclerotic inclination in coronary artery rather than the aorta and aorta root, the bounded atherosclerotic lesions as seen in the empirical LDLr^−/−^ and ApoE^−/−^ atherosclerosis mouse models, is subject to further explore.

## Conclusions

**Figure 10:**
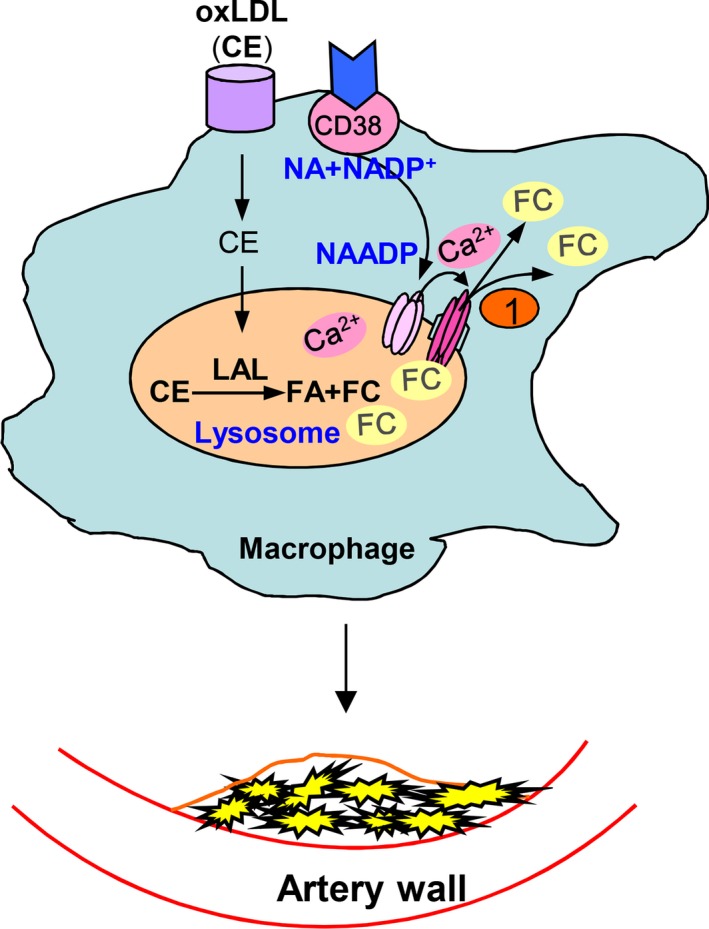
A work model showing CD38/NAADP Ca^2+^ signalling pathway in the regulation of lysosomal free cholesterol egression in the pathogenesis of atherosclerosis. The endocytosed oxLDL is trafficked into lysosomes where the cholesterol ester is hydrolysed to free cholesterol. The CD38 enzymatic product, NAADP, serves as a Ca^2+^ messenger to release Ca^2+^ from lysosomes. This local Ca^2+^ increase activates free cholesterol transporters (such as Niemann‐Pick type C1) and facilitates the egression of cholesterol from lysosomes. A deficiency in NAADP‐mediated Ca^2+^ release from lysosomes will lead to free cholesterol accumulation in lysosomes. This free cholesterol buildup will compromise lysosomal lumen acidity, Ca^2+^ storage and lysosomal acid lipase (LAL) activity; exacerbate cholesterol segregation in lysosomes in macrophages and result in atherosclerosis.

In summary, this study demonstrated that NAADP, a CD38‐derived lysosomal Ca^2+^ messenger, is essential for the free cholesterol efflux from lysosomes in mouse macrophages and that the deficiency of CD38 gene leads to lysosome free cholesterol segregation, lysosomal lipidosis and atherosclerosis, a work model with all major findings have been incorporated into a diagram (Fig. 10). To our knowledge, these findings provide the first experimental evidence indicating the critical role of CD38/NAADP signalling pathway in the pathophysiology of atherosclerosis. Understanding this important lysosomal signalling pathway in cholesterol metabolism and transportation may lend some novel therapeutic strategies for more efficient prevention and treatment of coronary atherosclerosis.

## Conflicts of interest

The authors confirm that there are no conflicts of interest.

## Supporting information


**Figure S1** Western blot assay confirmation of CD38 siRNA interference efficiency in macrophages. The summarized result showed that the expression of CD38 protein was significantly decreased (*P < 0.05 CD38 siRNA *versus* scrambled, *n* = 3).

**Figure S2** Enzymatic confirmation of the purity of lysosomal fractions. The purified lysosomes displayed a predominant enzymatic activity in acid phosphatase, a lysosome‐residing marker enzyme, but not in alkaline phosphatase and cytochrome C reductase, the marker enzymes of plasma membrane and endoplasmic reticulum, respectively, the two locations that are usually involved in cholesterol intracellular trafficking [*P < 0.05 compared with macrophage homogenates (Homogenate), *n* = 5].

**Figure S3** Microscopy images of oil red O–stained aorta and biochemical measurement of plasma cholesterol levels. (**A**) Oil red O–stained aorta atherosclerotic lesions (arrow) displayed only in LDLr^−/−^ mouse on WD, but not in both wild and CD38^−/−^ mice fed with either normal diet (ND) or WD (*n* = 5); (**B**) The overnight‐fasting lipid results showed that there were no significant differences in total and free cholesterol (Chol) levels in plasma from both wild‐type and CD38^−/−^ mice fed with either ND or WD for 12 weeks (*n* = 7).
