# Supplementary figures and images for: Lysosomal cholesterol accumulation in macrophages leading to coronary atherosclerosis in CD38−/− mice
Source: J Cell Mol Med. 2016 Jan 28;20(6):1001–13. doi: 10.1111/jcmm.12788 (PMC4882979; doi:10.1111/jcmm.12788)

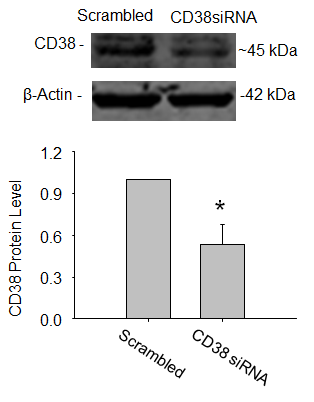

Supplement: Supplementary file 1 — Figure S1 Western blot assay confirmation of CD38 siRNA interference efficiency in macrophages. The summarized result showed that the expression of CD38 protein was significantly decreased (*P < 0.05 CD38 siRNA versus scrambled, n = 3). [file JCMM-20-1001-s001.tif]

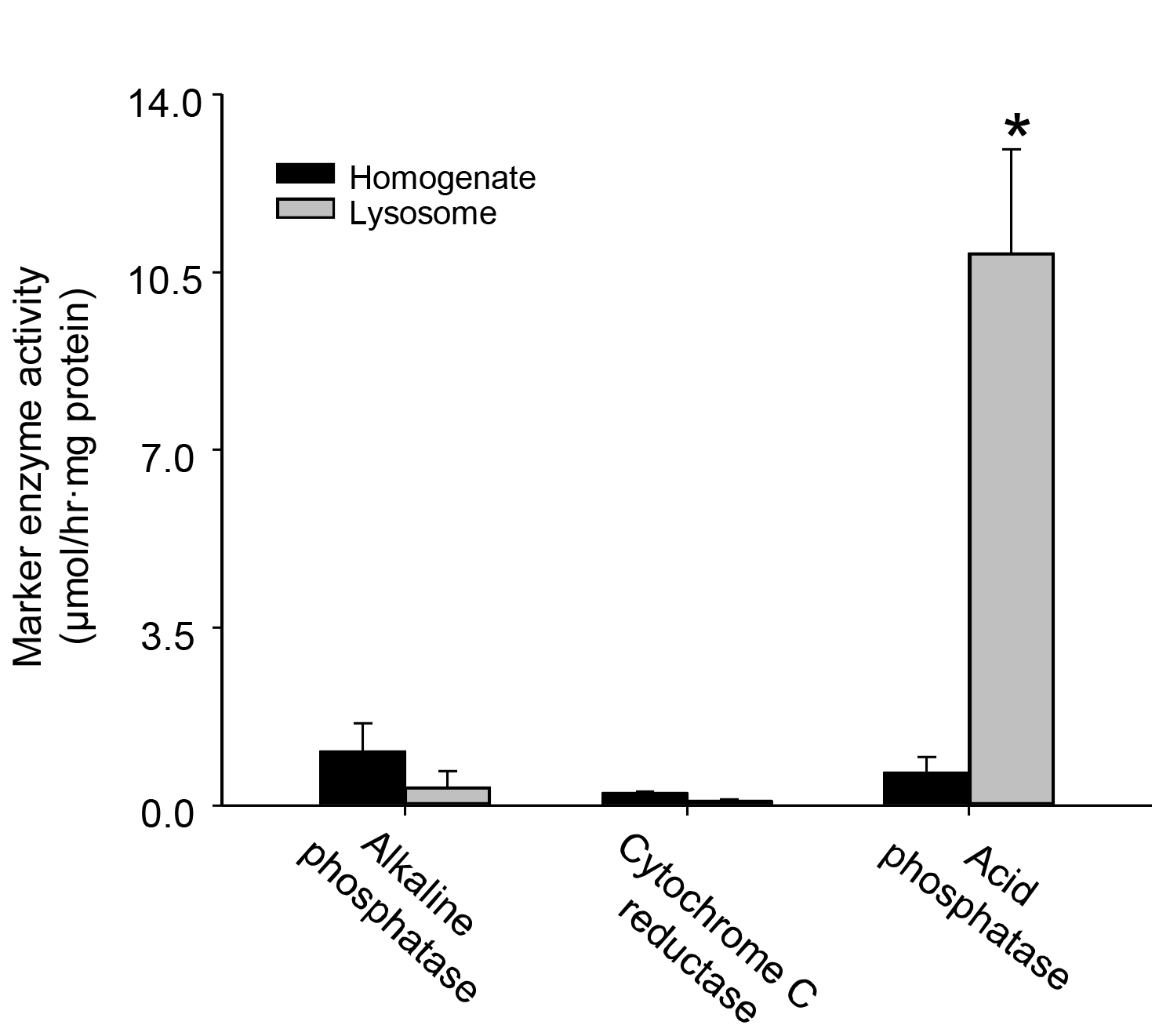

Supplement: Supplementary file 2 — Figure S2 Enzymatic confirmation of the purity of lysosomal fractions. The purified lysosomes displayed a predominant enzymatic activity in acid phosphatase, a lysosome‐residing marker enzyme, but not in alkaline phosphatase and cytochrome C reductase, the marker enzymes of plasma membrane and endoplasmic reticulum, respectively, the two locations that are usually involved in cholesterol intracellular trafficking [*P < 0.05 compared with macrophage homogenates (Homogenate), n = 5]. [file JCMM-20-1001-s002.tif]

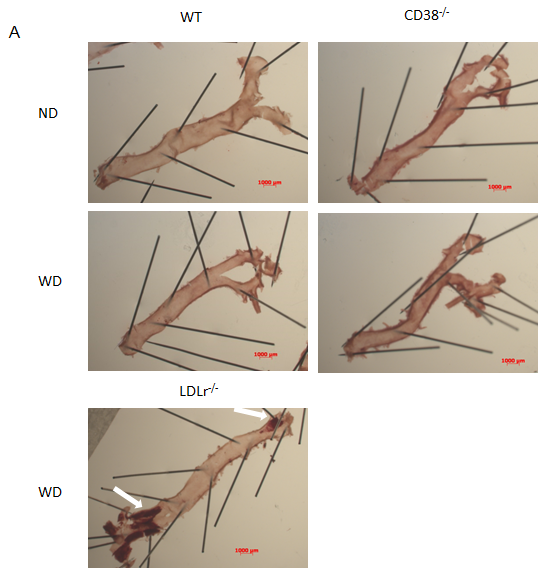

Supplement: Supplementary file 3 — Figure S3 Microscopy images of oil red O–stained aorta and biochemical measurement of plasma cholesterol levels. (A) Oil red O–stained aorta atherosclerotic lesions (arrow) displayed only in LDLr−/− mouse on WD, but not in both wild and CD38−/− mice fed with either normal diet (ND) or WD (n = 5); (B) The overnight‐fasting lipid results showed that there were no significant differences in total and free cholesterol (Chol) levels in plasma from both wild‐type and CD38−/− mice fed with either ND or WD for 12 weeks (n = 7). [file JCMM-20-1001-s003.tif]

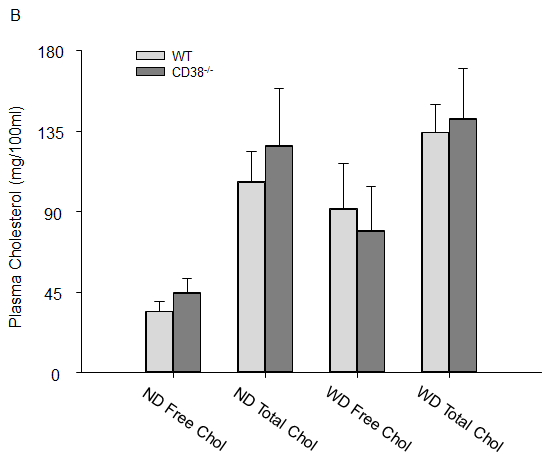

Supplement: Supplementary file 4 [file JCMM-20-1001-s004.tif]
